# The Transcriptome Landscape of Walnut Interspecies Hybrid (*Juglans hindsii* × *Juglans regia*) and Regulation of Cambial Activity in Relation to Grafting

**DOI:** 10.3389/fgene.2019.00577

**Published:** 2019-06-21

**Authors:** Qingguo Ma, Dechao Bu, Junpei Zhang, Yang Wu, Dong Pei

**Affiliations:** ^1^State Key Laboratory of Tree Genetics and Breeding, Key Laboratory of Tree Breeding and Cultivation of National Forestry and Grassland Administration, Research Institute of Forestry, Chinese Academy of Forestry, Beijing, China; ^2^Key Laboratory of Intelligent Information Processing, Advanced Computing Research Laboratory, Institute of Computing Technology, Chinese Academy of Sciences, Beijing, China

**Keywords:** *Juglans*, RNA-seq, *de novo* assembly, vascular cambium, graft

## Abstract

Walnuts (*Juglans*, Juglandaceae) are known throughout the world as economically important trees that provide fat, protein, vitamins, and minerals as a food source, and produce high-quality timber. We have amended the purpose section to say “However,” the omics resources are limited, which hampered the elucidation of molecular mechanisms resulting in their economically important traits (such as yield, fertility alternation, oil synthesis, and wood formation). To enrich the omics database of walnut, there is great need for analyses of its genomic and transcriptomic characteristics. In this study, we reported for the first time of the transcriptome landscape of six important organs or tissues in walnut interspecies hybrid using next-generation sequencing technology. Over 338 million clean reads were obtained. This yielded 74,072 unigenes with an average length of 782.71 bp. To develop an understanding of gene functions and regulatory pathways, 66,355 of the unigenes were identified as homologs of annotated genes and classified into three general categories with 61 functional subcategories. 2,288 out of 2,549 unmapped unigenes had at least one BLAST hit against the public databases. A total of 1,237 transcription factor-encoding genes (TFs) and 2,297 tissue-specific unigenes were identified. Interestingly, in the new shoot between an adult seedling and a grafted tree, the expression of 9,494 unigenes were significantly different, among which 4,388 were up-regulated and 5,106 were down-regulated. Of these, 195, 177, 232, 75, 114, and 68 unigenes were related to transcription factors, cell wall, defense response, transport, plant hormone biosynthesis, and other cambial activity-related functions, respectively. The obtained sequences and putative functional data constitute a resource for future functional analyses in walnut and other woody plants. These findings will be useful in further studies addressing the molecular mechanisms underlying grafting-related cambial activity.

## Introduction

Walnuts (*Juglans*, Juglandaceae) are among the most economically important trees cultivated in temperate regions all over the world and are valued for their nutritious nuts, high-quality wood, nut oil, and medicinal uses. In China, 487,007 ha were harvested for walnut production, and the production of walnuts (with shells) was 1,785,879 t (FAO, 2018), which equals 47.65% of the total world production. Nut-producing walnut cultivars are generally grafted onto rootstocks. In recent years, there has been increased attention on the use of interspecific hybrids as rootstocks. Offspring of California black walnut (*Juglans hindsii*) pollinated by Persian walnut (*J. regia*) is superior to its parents in several important traits (Potter et al., 2002; Li, 2017). This cultivar provides the rootstock for many commercial walnut trees. “Zhong Ning Sheng” (“ZNS;” *J. hindsii* × *J. regia*) is a cloned variety created through controlled pollination and propagation by cutting. Large-scale cultivation of “ZNS” in 11 provinces of China (50% of the suitable establishment area for walnut) have shown potential economic and ecological benefits. The characteristics of adaptation to a wide range of habitats, strong stress resistance, and well-developed root systems also make it a desirable species used for soil and water conservation and wasteland improvement (Li, 2017). Thus, the use of “ZNS” as a cultivar for rootstock, timber, and ecological applications is expanding. However, the mechanisms of regulation of its exclusive characteristics remain elusive due to the limited omics resources available. This is the first report on a walnut interspecies hybrid transcriptome. Further genomic studies and functional gene identification are necessary to uncover the genomic and transcriptomic characteristics.

“Zhong Ning Sheng” also demonstrates fast growth that produces high-quality timber, which makes it a superior rootstock for wood production (Li, 2017). In perennial woody plants, grafting is a simple and rapid technique for vegetative propagation. Various morphological and physiological characteristics change in response to grafting, including fruit quality, resistance, tolerance, and wood formation (Wang et al., 2017). The continuous increase in tree diameter over many years depends on the vascular cambium (VC) activity (Xu et al., 2016). It is believed that VC play key roles in determination of the vigor of tree growth (Begum et al., 2013; Zinkgraf et al., 2017b). Studies on the regulation of VC activity have always been a great concern (Costa et al., 2017; Reyes-Rivera et al., 2017; Kucukoglu et al., 2017). Cambium activity and the process of wood formation are also influenced by the sensitivity of cambium to plant hormones (Li, 2017; Li et al., 2017). Recent advances have shown that wood formation is regulated highly at the transcriptional level (Mizrachi and Myburg, 2016). A study of the comparative anatomy between new shoots from seedlings and grafted trees indicated significant differences. However, information on the molecular mechanisms involved in the various changes related to grafting is limited. Thus, differential expression analyses must be conducted to determine which regulatory factors are involved in the distinction between new shoots from seedlings and grafted trees.

Large-scale transcriptome sequencing using next-generation sequencing (NGS) technology enables the rapid determination of all transcripts for genome annotation and gene discovery (Jin et al., 2019; Khan et al., 2019). To uncover the walnut interspecies hybrid transcriptome landscape, RNA sequencing (RNA-seq) was performed on different organs and tissues of “ZNS.” Assembly based on transcriptional information from each sample was conducted to generate a more comprehensive transcript. The basic local alignment search tool (BLAST) was used to detect the distribution of gene ontology and sequence discrepancies. Based on extensive data analyses, we determined the expression profiles of tissue-specific RNAs. Diverse regulatory elements for VC activity changes related to grafting are also discussed. The results described here will assist in future research on gene discovery in walnut and other woody plants.

## Materials and Methods

### Plant Materials

Materials from five plant organs or tissues (roots, new shoots, leaves, female flowers, and immature fruit) from an “ZNS” adult seedling tree, and new shoots from a grafted 2-year-old “ZNS” tree maintained in Luoning County (Henan province, PRC) were used for the transcriptome landscape analysis. The sampled “ZNS” adult seedling tree was 23 years old and has been observed to consistently flower and bear fruit for years, and scions were taken to generate the grafted tree using the rootstock of a local walnut (*J. regia*). The six organs or tissues that were investigated were designated as root (RT), new shoot from “ZNS” adult seedling tree (MS), new shoot from grafted 2-year-old “ZNS” tree (JS), leaf (LF), female flower (FF), and immature fruit (IF). MS and JS were also used to study the transcriptional regulation of VC activity related to grafting, and were measured in triplicate.

Root was collected at the tillering stages. MS and JS were used as VC tissues, together with a small amount of its derivative tissues in the new shoots. They were collected simultaneously at the end of April when the activity of VC had begun. LF consisted of moderately mature leaflets collected from the middle part of the canopy on the sunny side. FF was collected at the pollination stage in spring. IF was collected after pollination once the fruit started to swell. To generate a more reliable transcriptome dataset, two mixed samples of all six organs or tissues were also used in this study. All of the samples were immediately frozen in liquid nitrogen stored at −80°C before to RNA isolation.

### RNA Isolation and Quality Control

Total RNA was isolated from plant materials with TRIzol kit (Invitrogen, CA, United States) and purified with RNase-free DNase I (Takara, Dalian, China). Extracted RNA was solubilized in 30 μL DEPC-treated H_2_O and monitored using 1% agarose gels to check the degradation and contamination. RNA purity was measured using the Nanodrop 2000 (Thermo Scientific, United States). A Qubit^®^RNA Assay Kit in Qubit^®^2.0 Flurometer (Life Technologies, CA, United States) was used to measure the RNA concentration and the RNA Nano 6000 Assay Kit of the Agilent Bioanalyzer 2100 system (Agilent Technologies, CA, United States) was used in RNA integrity assessment.

### cDNA Library Preparation and Sequencing

Qualified RNA was further purified by ribosomal RNA removal and used as input material for the subsequent cDNA library construction. Sequencing libraries were generated using NEBNext^®^Ultra^TM^ RNA Library Prep Kit for Illumina^®^(NEB, United States) and index codes were added to each sample for sequence attribution. Library fragments were purified with AMPure XP system (Beckman Coulter, Beverly, United States) with the purpose of selecting cDNA fragments of 150∼200 bp in length preferentially. Qualified cDNA was pre-processed with 3 μl USER Enzyme (NEB, United States) prior to PCR. PCR was performed and then the products were purified (AMPure XP system). Quality of constructed library was assessed using Agilent Bioanalyzer 2100 system.

The index-coded samples were clustered using TruSeq SR Cluster Kit v3-cBot-HS (Illumina) on a cBot Cluster Generation System. and sequenced on an Illumina Hiseq 2500 platform (Novogene Bioinformatics Technology Co., Ltd., Beijing, China) and 125 bp paired reads were generated. All datasets have been submitted to NCBI Sequence Read Archive (SRA) database and the files can be found under the accession number SRR8240489-SRR8240495. Assembled sequences together with sequence annotation can be achieved from figshare^1^. Sequencing depth for each of the individual samples and their biological replicates is 5G while 15G and 20G separately for other two mixed samples.

### Reads Mapping and Transcriptome Assembly

Adaptor sequences, low quality reads and reads less than 50 bp in length were omitted using Trimmomatic^2^. Subsequently, the remaining reads were mapped using the SILVA rRNA database with BLAST software (-evalue 1e-40 -min_raw_gapped_score 110 -perc_identity 95.0). Filtered reads were aligned against the *J. regia* genome sequences^3^ (Martínez-García et al., 2016) and then assembled into contigs using Trinity (Borodina et al., 2011; Grabherr et al., 2011) with a *de novo* assembly strategy (–normalize_reads –normalize_max_read_cov 100 –min_kmer_cov 2 –KMER_SIZE 32).

### Annotations With Different Databases and Identification of Transcription Factors

The sequences of the assembled transcripts were compared against NCBI Refseq Plants^4^, Swiss-Prot (The UniProt Consortium, 2017), eggNOG (Huerta-Cepas et al., 2016), Gene Ontology (GO) (Ashburner et al., 2000), Kyoto Encyclopedia of Genes and Genomes (KEGG) (Kanehisa and Goto, 2000), TAIR10 protein database (Lamesch et al., 2012), and Pfam (Finn et al., 2016) using the software packages Trinotate 3.0^5^ (Bryant et al., 2017), TransDecoder 3.0 (Grabherr et al., 2011), Blast 2.6.0 (blastx/blastp -evalue 1e-5; blastn -evalue 1e-20) (Lobo, 2012) and HMMER3 (Finn et al., 2011) according to the user manual and excellent hits were processed for functional annotation. Homologs were sequentially annotated according to the blast results.

Gene ontology analysis was performed using Gene Ontology annotations tool^6^ available at TAIR. KO (KEGG Orthology) IDs were obtained from KAAS (KEGG Automatic Annotation Server^7^) using the TAIR IDs assigned to unigenes.

We aligned the transcription factors (TFs) to the plant transcription factor database (PlantTFDB) available at http://planttfdb.cbi.pku.edu.cn (Jin et al., 2017). The identified TFs were subsequently classified into corresponding families.

### Tissue-Specific mRNA Expression Profiles, Functional Analysis, and qRT-PCR Validation

Expression profiles of tissue-specific RNAs were quantified and evaluated using an entropy-based method (Song et al., 2016). GOs and KEGGs were enriched for each tissue-specific expressed set to predict their involved functions.

Gene-specific primer sets were designed and relative real time PCR (qRT-PCR) was performed in triplicates with the same plant materials using the SYBR Premix Ex Taq^TM^ II Kit (Takara, Dalian, China) on a Roche light Cycler 480 (Roche Applied Science, Penzberg, Upper Bavaria, Germany) to validate the expression of tissue-specific RNAs. The 2^−ΔΔCT^ method was conducted to determine the relative copy number of genes based on the qRT-PCR data (Livak and Schmittgen, 2001).

### Statistical Analyses

Expression levels of all unigenes/transcripts were analyzed using the RSEM v1.3.0 (Li and Dewey, 2011) using transcripts per million (TPM). Unigenes with a cut-off value of TPM > 3.0 in any of the investigated organs or tissues were selected. The Bioconductor edgeR package was used for DEG analysis (Robinson et al., 2010). Samples were normalized based on trimmed mean of *M*-values (TMM) and thresholds of gene expression difference significance were set on the co-occurrence of absolute value of logFC (fold change)>1.5 and *P* < 0.05. qRT-PCR data are averaged from three biological replicates and are expressed as mean ± standard deviation (SD).

## Results

### The “ZNS” Transcriptome Landscape

#### RNA-Seq and *de novo* Assembly

**FIGURE 1:**
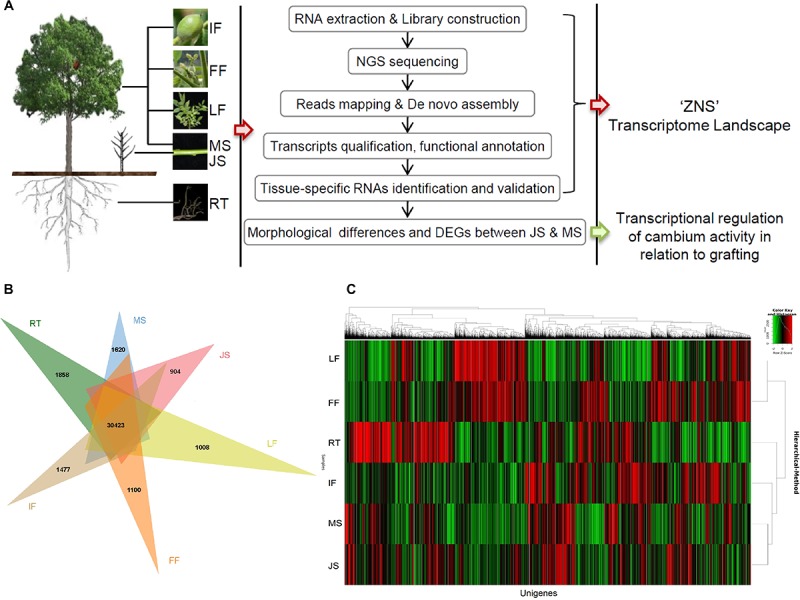
Overview of the experimental design and the general picture of “ZNS” transcriptome landscape. **(A)** Experimental protocol. **(B)** Distribution of unigenes expressed in different organs/tissues. **(C)** Hierarchical clustering analysis of unigenes expressed in all six libraries. RT, root; MS, new shoot of adult “ZNS” seedling tree; JS, new shoot of grafted “ZNS” tree; LF, leaf; FF, female flower; IF, immature fruit.

To investigate the walnut interspecies hybrid transcriptome landscape, cDNA libraries were prepared from mRNA from each of the different organs or tissues of “ZNS,” and these were used for sequencing using Illumina NGS technology (Figure 1). To generate a more reliable transcriptome dataset, two mixed samples from all six organs or tissues were also used in this study to produce much deeper sequence depths. Transcriptome data revealed a high similarity between biological triplicates with a Spearman correlation of 0.9187. In total, 356,073,415 raw paired-end reads (approximately 82.91 Gbp) were trimmed from which adaptors were filtered and low-quality sequences were removed. The remaining reads were mapped using the SILVA rRNA database and 4,306 rRNA reads were removed. A total of 338,269,744 (approximately 78.77 Gbp; 95% of the raw data) high-quality clean reads with 96.23% of Q20 plus bases were obtained (Table 1 and Supplementary Figure S1). The mapped reads ratio against the latest version of the published *J. regia* genome^8^ (Martínez-García et al., 2016) averaged 86.03%. Subsequently, a *de novo* assembly strategy was conducted based on the high-quality filtered sequence data using Trinity (Borodina et al., 2011; Grabherr et al., 2011). As a result, 448,288 contigs were generated totally. The contig length was averaged 811.06 bp and the N50 contig size of the assembled data was 1,217 bp. 74,072 non-redundant sets of unigenes were formed by pooling these contigs together (Table 1).

**Table 1:** Summary statistics for the sequencing and *de novo* assembly of “ZNS” transcriptome.

Category	Value
Total number of clean reads	338,269,744
Total clean nucleotides (bp)	84,575,420,575
Q20 percentage (%)	96.23
Total trinity “genes”	74,072
Total trinity transcripts	127,617
Genes with multiple-isoform	22,247
N50 contig size^a^ (bp)	1,217
Average contig size (bp)	811.06

*^a^ N50 corresponds to the length of the smallest contig in the set of largest contigs whose combined length represents 50% of the total assembly size.*

Expression levels of all unigenes/transcripts were analyzed using the RSEM v1.3.0 (Li and Dewey, 2011) using TPM. The abundance of unigenes/transcripts expressed (cut-off value, TPM > 3.0) in all six of the investigated organs or tissues were relatively similar. However, a much lower unigene/transcript abundance was observed for female flowers (FF; 46,671, 63.01%). The largest transcriptome size was obtained from new shoots from the “ZNS” adult seedling tree (MS; 52,771, 71.24%), followed by new shoots from the grafted 2-year-old “ZNS” tree (JS; 52,115, 70.36%). The numbers of assembled unigenes/transcripts in the leaf (LF; 50,619, 68.34%) and immature fruit (IF; 50,426, 68.08%) samples were very similar, whereas those detected in the roots (RT; 47,631, 64.30%) were comparatively low (Figure 1B). The expression patterns of the 30,423 shared unigenes in different organs or tissues are shown in Figure 1C, which indicates a complex global view of gene expression in “ZNS.”

Subsequently, an analysis of the abundance of 458 euKaryotic Orthologous Groups (KOGs) and 248 ultra-conserved core eukaryotic genes (CEGs) using the Core Eukaryotic Gene Mapping Approach (CEGMA) pipeline was conducted to evaluate the completeness of this transcriptome library (Kenkel and Bay, 2017). A comparison analysis revealed the presence of 455 (99.34%) out of 458 KOGs in the assembly. The orthologous proteins in the hit-unigene against the CEG database would be useful to studies on protein classification and evolutionary rates research (Garg et al., 2011). Analysis using CEGMA predicted 246 (99.19%) of the 248 highly conserved core proteins in the assembly, indicating the integrity of the “ZNS” transcriptome in this study was very well (Supplementary Table S1).

#### Homology Searches and Functional Annotation

In addition to the KOG database, gene annotation was performed against different databases based on sequence homologies. Of 74,072 unigenes, 66,355 (89.58%) were matched within these databases (Table 2). Similarity between 63,348 (85.52%) of the annotated unigenes/ transcripts and the known sequences exceeded 80%, with alignments of over 200 bp in length for 27.51% (20,377 unigenes) of the annotated sequences (Supplementary Table S2). 75,413 out of 127,617 transcripts were identified as coding sequences (CDSs) using BlastX and ESTscan software, with mapping lengths over 296 bp and an average mapping length of 707.6 bp. As for transcripts that were not classified into CDSs, either because their coding regions did not meet the criteria for CDS prediction, or because they may have been non-coding RNAs (Liu et al., 2017).

**Table 2:** Annotation of “ZNS” unigenes in different databases.

Database	Number of annotated unigenes
Swiss-Prot	50,978
Refseq Plant	66,515
Refseq Plant (ncRNA)	53,608
Pfam	32,564
Gene ontology	49,779
KEGG	44,861
COG	43,230
Total annotated unigenes	66,355

Gene ontology is a popular standard in gene annotation and provides a structured vocabulary for describing gene products. Unigenes were mapped to transcriptomes and genomes of related plant species separately using BLAST software (*E*-value threshold of 1.0E-5). A filter strategy (-minId = 0.9, -minAlnSize = 50, -minQSize = 2 00, -globalNearBest = 0, -minNonRepSize = 16, -ignoreNs, -bestOverlap) was then conducted using the pslCDnaFilter program. Annotations from transcriptomes and genomes were merged and the redundant information was eliminated.

**FIGURE 2:**
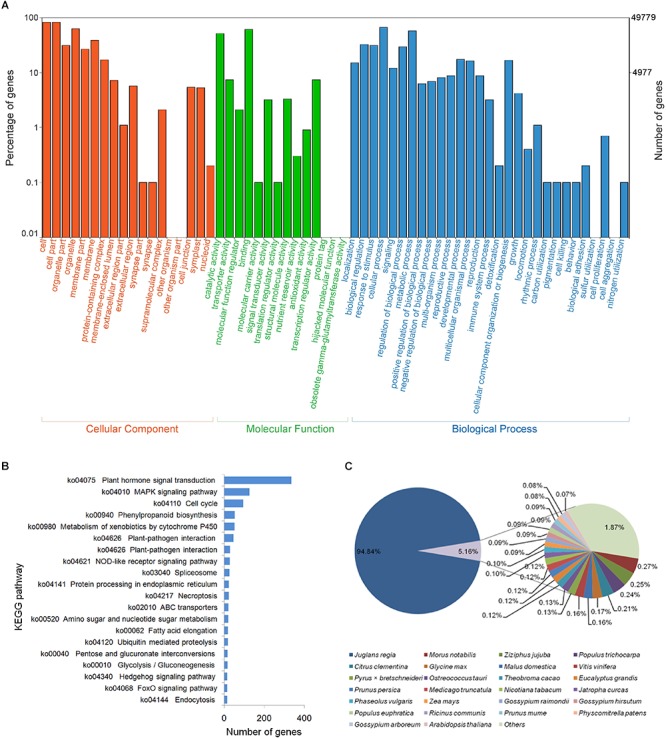
Overview of gene annotation against the public databases for “ZNS” unigenes based on RNA-seq data. **(A)** Gene Ontology (GO) function category by BLASTx with an *E*-value threshold of 10^−5^. **(B)** Top 20 identified Kyoto Encyclopedia of Genes and Genomes (KEGG) pathways. **(C)** Species distribution of the top BLASTx hits.

As a result, 86,697 transcripts (49,779 unigenes, 67.20%) were assigned GO annotations, classified into biological process, cellular component, and molecular function with a ratio of 32.30, 34.40, and 33.29%, respectively (Figure 2A). Of the unigenes sorted to a biological process, 33,195 (66.70%) and 28,615 (38.34%) were concentrated in cellular processes and metabolic processes, respectively. For the cellular component category, 82.70%of the unigenes were active participants in the cell and 82.50% for cell parts, whereas 62.80% of the unigenes were for organelles. There were 14 subcategories for molecular function, including 30,361 (61.00%) unigenes that were identified as involved in “binding.” Several “activity” terms were identified, such as catalytic activity (51.50%), transporter activity (7.40%), and transcription regulator activity (7.40%). This may indicate that fast growth and a mass of metabolic activities were continuing in the analyzed organs or tissues.

In addition, pathway analysis was carried out using KEGG for all of the assembled unigenes. Of the unigenes, 60.56% (44,861) were mapped to the KEGG pathways and 14,807 metabolic pathways were predicted (Figure 2B). The major portion of the classified unigenes were categorized as plant hormone signal transduction (Ko04075, 336 sequences), followed by the MAPK signaling pathway (Ko04010, 126 sequences), and cell cycle (Ko04110, 95 sequences). These are important pathways that represent the majority of the metabolic processing in walnut interspecies hybrid, in consideration of the samples used in this study came from the primary tissues or organs. This indicates that the data will serve as an invaluable resource for further studies on functional genomics in the genus *Juglans*.

In the databases, the top-hit species was *J. regia* (94.84%), followed by *Morus notabilis* (0.27%), *Ziziphus jujuba* (0.25%), and *Populus trichocarpa* (0.24%), whereas 0.07–0.21% of the annotated unigenes were distributed in *Citrus clementine*, *Glycine max*, *Malus domestica*, *Vitis vinifera*, *Pyrus* × *bretschneideri*, *Ostreococcus tauri*, *Theobroma cacao*, *Eucalyptus grandis*, *Prunus persica*, *Medicago truncatula*, *Nicotiana tabacum*, *Jatropha curcas*, *Phaseolus vulgaris*, *Zea mays*, *Gossypium raimondii*, *Gossypium hirsutum*, *Populus euphratica*, *Ricinus communis*, *Prunus mume*, *Physcomitrella patens*, *Gossypium arboreum*, and *Arabidopsis thaliana* (Figure 2C).

The transcriptome data was compared with the *J. regia* genome information. As a result, 71,523 unigenes (96.56% of the assembled unigenes) were mapped to the genome scaffolds, while 2,549 unigenes (3.44%) were not. A total of 89.76% (2,288 unigenes) of the unmapped unigenes had at least one BLAST hit against the public databases. Nucleus (GO:0005634), structural constituent of ribosome (GO:0003735), and integral component of membrane (GO:0016021) were among the top three GO hits. The number of unmapped unigenes in each of the six organs or tissues varied. The most tissue-specific unmapped RNAs were found in RT (161 unigenes), whereas 32, 38, 11, 18, and 25 unigenes were found in MS, JS, FF, LF, and IF, respectively.

#### Transcriptome-Wide Identification of TFs

**FIGURE 3:**
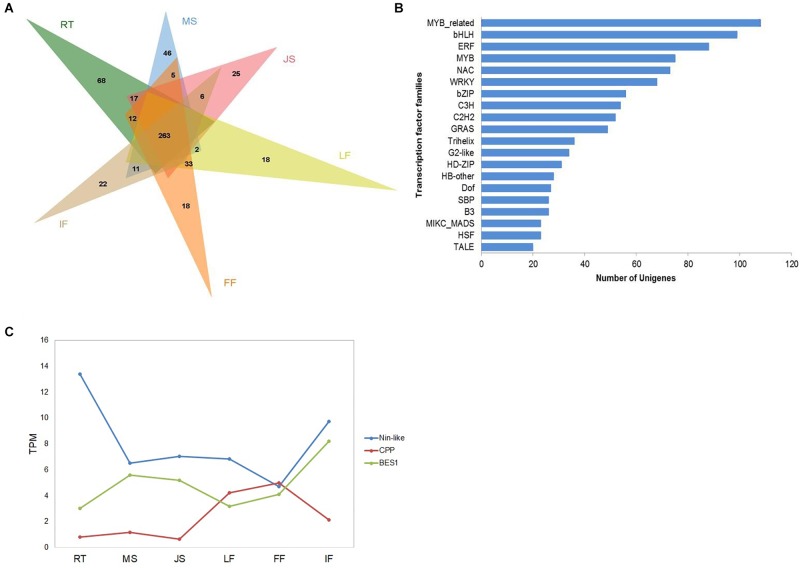
Overview **(A)**,distribution of top 20 identified transcription factors **(B)**, and expression patterns of several selected TFs **(C)** from “ZNS” unigenes into transcription factor families. RT, root; MS, new shoot of mature “ZNS” tree; JS, new shoot of grafted “ZNS” tree; LF, leaf; FF, female flower; IF, immature fruit. MYB-related (103 unigenes), bHLH (99 unigenes), and ERF (88 unigenes) were among the top three families.

Transcription factors play important roles in regulating gene expression. Among the walnut interspecies hybrid unigenes, totally 1,237 TFs were identified. Of the 1,237 TFs, 263 TFs were expressed in all analyzed organs or tissues, whereas 68, 46, 25, 18, 18, and 22 TFs were specifically expressed in RT, MS, JS, LF, FF, and IF, respectively (Figure 3A). All of the TFs were further classified into 52 families (Figure 3B). MYB-related (103 unigenes), bHLH (99 unigenes), and ERF (88 unigenes) were among the top three families. Y-subunit, GRAS, E2F, X1, and bHLH TF families were also present. In general, 84.62% of the identified TFs were found in all six organs or tissues, whereas WRKY, YABBY, LSD, SRS, CPP, GRF, RAV, and Nin-like TFs were differentially expressed. LSD TFs were exclusively expressed in RT, MS, JS, FF, and IF. SRS TFs and Nin-like TFs were differentially expressed in RT, LF, FF and IF. WRKY TFs were exclusively expressed in RT, MS, JS and IF. YABBY TFs, CPP TFs and GRF TFs were exclusively expressed in LF, FF, and IF. RAV TFs were exclusively expressed in RT and IF. BES1 TFs, major transcription factors in BR (brassinosteroid) signaling that play roles in regulating plant growth, development, and stress resistance (Wang et al., 2018), were found to be expressed in all six organs or tissues (Figure 3C).

#### Tissue-Specific mRNA Expression Profiles and Sequence Validation

**FIGURE 4:**
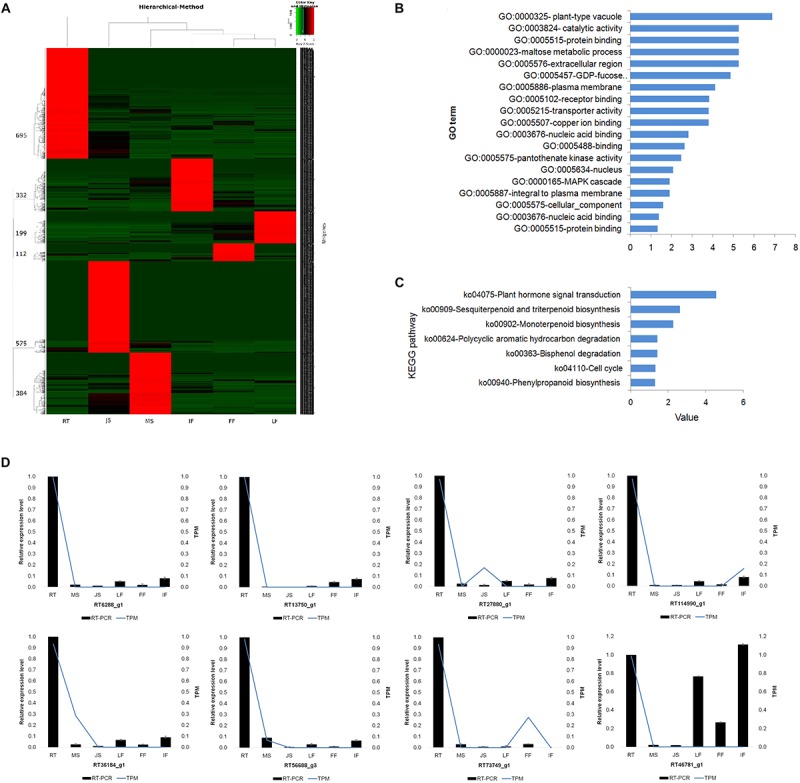
Identification, functional analysis and validation of tissue-specific expressed unigenes in 6 different organs/tissues. **(A)** Distribution and hierarchical cluster analysis of tissue-specific expressed unigenes. **(B)** GO enrichment of specific expressed genes in JS. **(C)** KEGG enrichment of specific expressed genes in JS. **(D)** Exhibition for the abundance of unigenes expressed specifically in Root validated by relative qRT-PCR (see complete results in Supplementary Figure S2). RT, root; MS, new shoot of adult “ZNS” seedling tree; JS, new shoot of grafted “ZNS” tree; LF, leaf; FF, female flower; IF, immature fruit. Curves showing the expression level of transcriptomic sequencing while bars showing the results of qRT-PCR. Error bars represent mean ± standard deviation (*SD*) and the data are averages from three biological replicates.

Jensen-Shannon tissue specificity score (JS score) (Altschul et al., 1990) was employed and calculated for each transcript to assess the tissue-specificity of the expression of the RNAs. Using a JS score of 0.9 as a cut-off, we identified 2,297 (3.10%) tissue-specific unigenes. The numbers of detected tissue-specific unigenes revealed dramatic differences between the six organs or tissues. RT exhibited the most tissue-specific unigenes (695, 30.26%), whereas FF demonstrated the lowest number (112, 4.88%). Thus, the expression of some of the genes was clearly subject to tissue-dependent regulation (Figure 4A).

Gene ontology and KEGG enrichments were conducted to analyze the roles of the unigenes specifically expressed in each organ or tissue (Figure 4B,C). For example, the unigenes specifically expressed in MS mainly involved proteins such as xylem cysteine proteinase 2 (XCP2) precursor (MS73240_g1), Os4bglu 12 β-glucosidase (MS32674_g1), glycerophosphoryl diester phosphodiesterase (GDPD) family protein (MS26356_g1), and pectate lyase (PEL) precursor (MS7907_g1).

The relative qRT-PCR was performed for 16 randomly selected tissue-specific unigenes in RT to validate the RNA-seq results. All reactions generated sequence products (Figure 4D). The relative qRT-PCR measurements were positively correlated with the RNA-seq results for 15 (93.75%) of the tested genes (*R*^2^ = 0.934, *P* < 0.01). The qRT-PCR results agree well with RNA-seq analyses which also validated the accuracy of *de novo* assembled transcriptome and tissue-specific RNAs analysis in the present study.

### Transcriptional Regulation of Cambium Activity Related to Grafting

#### Morphological Changes in VC From Seedling and Grafted “ZNS” Tree

**FIGURE 5:**
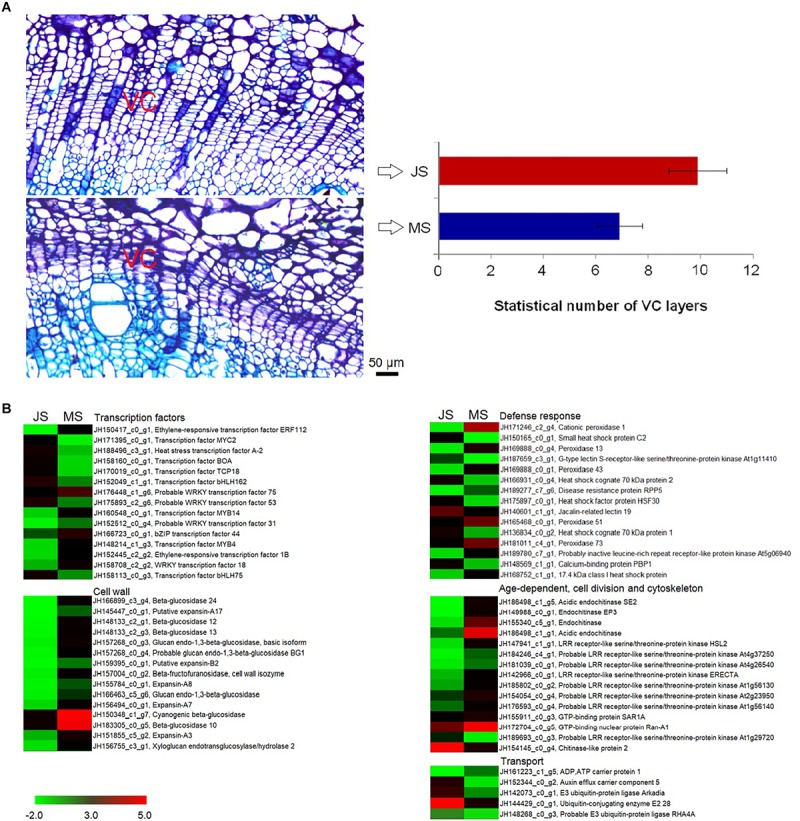
Morphological and transcriptional changes of VC activity between MS and JS. **(A)** VC morphology differences between 2 samples. **(B)** Heatmap of differentially expressed genes (logFC > 1.5) involved transcription factors, defense response, age-dependent, cell division, cytoskeleton, cell wall, and transport (the complete results are shown in Supplementary Figure S3). The bar represents the scale of the expression levels for each gene (TMM) in the MS and JS as indicated by red/green rectangles. Red rectangles indicate up-regulation of genes and green represents down-regulation. VC, vascular cambium; MS, new shoots from “ZNS” adult seedling tree; JS, new shoots from “ZNS” grafted tree.

Morphological differences in VC between the JS and MS tissues were investigated. Cell layers within VC was counted using imaging (Figure 5A). Both samples were investigated at the end of April, approximately 30 days after the VC began dividing. For JS, the cambial zone of the shoot consisted of approximately 9.90 ± 1.09 layers of cells containing dense cytoplasm, whereas for MS, the cambial zone had around 6.90 ± 0.88 layers of cells that showed distinct differences (*P* < 0.05). Thus, a cytological foundation was provided for further studies on the molecular mechanism underlying cambial activity related to grafting.

#### Differentially Expressed Unigenes Involved in VC Activity Change

Significant differences in gene expression related to VC activities between JS and MS were identified (*P* < 0.05, logFC > 1.5). The expression of 9,494 genes changed significantly between these two samples, of which 4,388 were upregulated and 5,106 were downregulated, indicating that transcript abundance dramatically changed after grafting. The significantly enriched annotations demonstrated that there were 195, 177, 232, and 75 unigenes related to transcription factors, cell wall, defense response, and transport, respectively. Moreover, 68 unigenes involved age-dependent, cell division-related, cytoskeletal, and other VC activity-related functions. Several unigenes’ expression differed significantly (logFC > 4), which included the genes encoding transcription factors (ethylene-responsive transcription factor ERF112 and MYB4), cell wall-related proteins (such as beta-glucosidase 12, expansin-A1, glucan endo-1,3-beta-glucosidase, probable xyloglucan endotransglucosylase/hydrolase protein 32, and probable glucan endo-1,3-beta-glucosidase BG1), and defense response proteins (such as cationic peroxidase 1, heat shock cognate 70 kDa protein, peroxidase 13, and G-type lectin S-receptor-like serine/threonine-protein kinase LECRK1) (Figure 5B).

**FIGURE 6:**
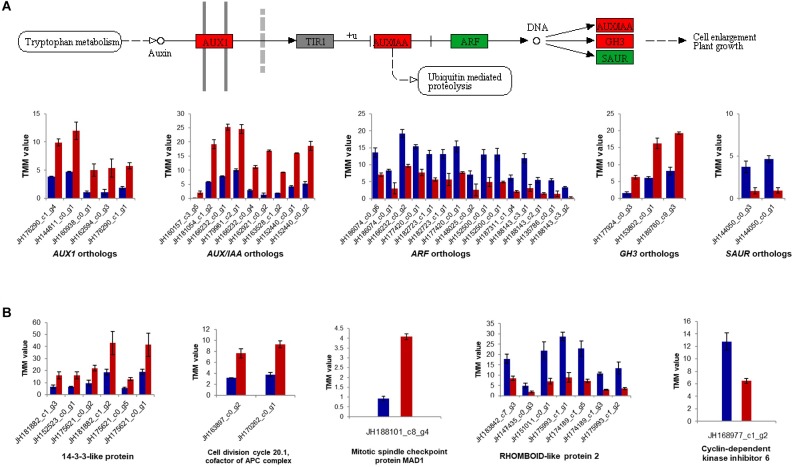
Expression patterns for genes involved the plant hormone signal transduction pathway (Ko04075) and the cell cycle pathway (ko04110). **(A)** Auxin signal transduction pathway and relative expression of differentially expressed genes. **(B)** Relative expression of differentially expressed genes involved the cell cycle pathway. TMM, trimmed mean of *M*-values. Red, green, and gray squares showing up-regulated, down-regulated and undetected genes, separately. Red bar shows the sample of new shoots from “ZNS” grafted tree (JS) and blue bar shows new shoots from “ZNS” adult seedling tree (MS). Error bars represent mean ± standard deviation (SD) and the data are averages from three biological replicates. The complete results are shown in Supplementary Figures S4, S5.

Subsequently, global changes in plant hormone-related genes between MS and JS of “ZNS” were identified (Figure 6). Some genes involved in the plant hormone signal transduction pathway (Ko04075), including auxin transporter-like proteins (*JH176290_c1_g4*, *JH144811_c0_g1*, *JH160938_c0_g1*, *JH162594_c0_g3*, and *JH176290_c1_g1*), auxin-induced proteins (*JH160157_c3_g5*, *JH181054_c1_g2*, *JH166232_c0_g1*, and *JH179961_c2_g1*), and probable indole-3-acetic acid-amido synthetase proteins (*JH188245_c1_g1*, *JH164588_c0_g1*, *JH177924_c0_g3*, *JH153802_c0_g1*, *JH189760_c9_g3*, *JH170722_c0_g1*, and *JH170722_c0_g2*) were downregulated. Histidine kinase 4 genes (*JH135974_c2_g1*, *JH188244_c4_g5*, *JH144766_c0_g3*, *JH188306_c2_g6*, *JH188306_c2_g5*, *JH133044_c2_g1*, *JH188244_c4_g4*, and *JH188306_c2_g4*) were downregulated in response to CTK as well. In addition, abscisic acid receptor PYL4 (*JH160657_c0_g1*) was found to be upregulated in the new shoot from “ZNS” grafting (Figure 6A). Several other genes, such as auxin transporter protein (*JH187416_c2_g4*), auxin-responsive proteins (*JH182597_c1_g4*, *JH166232_c0_g4*, *JH144782_c0_g2*, *JH159582_c1_g2*, *JH183781_c1_g3*, *JH182597_c2_g2*, *JH153865_c0_g2*, *JH144050_c0_g3*, and *JH144050_c0_g1*), and auxin response factors (*JH148025_c0_g2*, *JH152500_c0_g1*, *JH152500_c0_g1*, *JH187311_c1_g4*, *JH188143_c3_g1*, *JH188143_c2_g1*, *JH135786_c0_g1*, *JH188143_c3_g2*, *JH186074_c0_g6*, *JH186074_c0_g1*, *JH166232_c0_g2*, *JH177420_c0_g1*, *JH182723_c1_g1*, *JH182723_c1_g1*, *JH177420_c0_g1*, *JH137485_c0_g2*, *JH143832_c0_g1*, *JH137485_c0_g1*, and *JH135087_c1_g1*) were downregulated in the new shoot from the grafted “ZNS” tree. These observations provide further insight into the role of plant hormones in VC cell development and wood formation.

Some genes involved in the cell cycle pathway (Ko04110), such as 14-3-3 protein 7 (*JH157469_c0_g2*), 14-3-3-like proteins (*JH181882_c1_g3*, *JH152523_c0_g1*, *JH175621_c0_g2*, *JH181882_c1_g2*, *JH175621_c0_g5*, *JH175621_c0_g1*, *JH181299_c0_g4*, *JH157469_c0_g1*, and *JH174101_c2_g1*), cell division cycle 20.1 (*JH163897_c0_g2* and *JH170262_c0_g1*), MCM5 (*JH183969_c2_g1*), and MAD1 (*JH188101_c8_g4*) were upregulated in JS, as expected. However, cyclin-dependent kinase inhibitor 6 (*JH168977_c1_g2*) and rhomboid-like protein 2 (*JH183842_c7_g3*, *JH147435_c0_g3*, *JH151011_c0_g1*, *JH175993_c1_g1*, *JH174189_c1_g5*, *JH174189_c1_g3*, and *JH175993_c1_g2*) were downregulated (Figure 6B).

## Discussion

### Detailed Transcriptome Dataset for the Study of Interspecies Hybrids

Cross-breeding is an important method for the genetic improvement of woody plants. Heterosis, also known as hybrid vigor, is a widespread trait in plants and animals (Chen, 2013). It is not well understood but has been exploited extensively in breeding and for commercial purposes. Studies of the molecular basis of heterosis have focused on the super hybrids produced by cross-breeding. However, basic datasets generated from hybrids are limited and the molecular and genetic mechanisms underlying the phenotypic changes in hybrids remain poorly understood. The transcriptional data from an interspecies hybrid that serves as a common rootstock are expected to be more relevant than previous data, and provide insight into the evolutionary mysteries of heterosis. The present study aimed to enhance the genetic resources available for walnut and other perennial woody plants by providing a transcriptome landscape obtained using NGS technologies, which is powerful and relatively inexpensive (Linde et al., 2015; Briese et al., 2016; Leng et al., 2017).

In this study, up to 356 million 125-bp paired-end reads and 74,072 assembled unigenes with an average length of 782.71 bp were obtained using the Illumina HiSeq 2500 platform. Considering the low mapping rate to the published genome for *J. regia*, which served as the male-parent species of “ZNS,” a *de novo* assembly strategy was utilized. *De novo* assembly of RNA-seq data allows the study of transcriptome in absence of a reference genome. Ineluctably, *de novo* assembly of transcriptome may lead to a few technical errors because there may be multiple isoforms for each gene due to alterative splicing. Raw reads were abundant and strictly filtered before *de novo* assembly, so our transcriptome sequences were of relatively high quality. Mapping of unigenes against KOG and CEG proteins at very low (10^−10^) *e*-values indicates the quality and integrity of the sequencing and assembly is relatively high. We found several sequences show no similarities with the reference genome, which may be unique sequences inherited from male parent or potential walnut hybrid-specific sequences. Thus, a high-resolution transcriptome atlas of the walnut interspecies hybrid has been constructed. In this study, the transcriptome size varied moderately between the different organs or tissues. The possibility of variability in transcriptome size in different organs or tissues in the walnut interspecies hybrid is demonstrated in the present study.

### Functional Annotation of Unigenes and Tissue-Specific Expression Profiles

Gene ontology and KEGG were further employed to carry out functional annotation and classification of annotated unigenes. Functional annotation of these unigenes includes functions covering almost all biological processes. In this research, the transcripts most highly expressed in MS involved the xylem cysteine protease 2 (XCP2) precursor, whereas the most highly represented unique transcripts in the remaining five organs or tissues were involved in metal cation transporter, CCT motif family protein, transferase family protein, amino transferase, and histidine acid phosphatase. XCP2 precursor is involved in tracheary element differentiation (Altermann et al., 2005). Other than XCP2 precursor, os4bglu 12 β-glucosidase and pectate lyase (PEL) precursor were also found to be significantly expressed in MS. Os4bglu 12 β-glucosidase is a family 1 glycoside hydrolase that was found to possess high exoglucanase activity and may play an important role in defense, as well as in cell wall-derived oligosaccharide breakdown (Baiya et al., 2018). Pectate lyase (PEL) precursor was found to be involved in the maintenance of normal cell division (Opassiri et al., 2010). These results indicate that the abundance of transcripts has different characteristics in different tissues, which reflects the unique transcriptome characteristics in different tissues.

Annotated unigenes were further clarified into 319 significant KEGG pathways. Among these, carbon metabolism, biosynthesis of amino acids, and plant hormone signal transduction had higher representation. Furthermore, several unigenes represented no hits in BLAST analyses, the majority of which may be non-coding RNAs, sequences of uncharacterised genes, or potential walnut hybrid-specific genes. In general, *de novo* transcriptome sequencing data presented here is an important source of transcriptome sequence information for functional research in the walnut interspecies hybrid or other tree species, and also confirm that NGS technology is avaluable tool for transcriptome characterisation and gene discovery in non-model woody plants.

### Genome-Wide Expression Profiling Revealed Gene Regulation Modules Involved in VC Activity Changes Related to Grafting

Secondary growth, which relies on the activity of meristems, plays an important role in tree biology. VC activity affects the growth of trees, including wood properties (Woerlen et al., 2017). Grafting is one of the most important techniques used for the reproduction of trees to maintain the plant’s desirable characteristics. Many species of trees are dependent on grafting for reproduction. In the “ZNS” seedlings and grafted trees, the cytological morphology of the shoots revealed significant differences, indicating that the growth patterns differed significantly due to grafting. To analyze the factors regulating the VC activity at transcriptome level, we carried out an integrated transcriptional analysis of genes involved in grafting-related differences. Comparisons of the gene expression between these two samples suggested that the comprehensive effects of plant hormone pathways and cell cycle pathway, cell wall, and cell division may be involved in the regulation of VC activity. Significant differential expression was found for several transcription factor family members related to meristem function and they may be important in the vascular system development (Hugouvieux et al., 2018). For example, WRKYs are key regulators of various plant processes and regulates responses to the hormone abscisic acid (ABA). MYBs play key roles in regulatory networks controlling vascular differentiation in plants. In the present study, these TFs were found to be differentially expressed. These findings reveal developmental modules that are related to differences in growth patterns and provides insights into the network regulating the VC activity related to grafting in woody plants.

Wood formation is largely regulated by a number of plant hormones (Zinkgraf et al., 2017a). To understand growth alterations related to grafting in perennial woody plants, these plant hormones should be taken into account in genetic analyses. AUX/IAA proteins play important roles in auxin signaling (Zhao et al., 2019). The present study reveals that several transcripts for proteins that are involved auxin and ABA signal responses were differentially expressed in the investigated tissues, such as 14-3-3-like protein and auxin-regulated protein.

## Conclusion

The present study reveals for the first time the complexity of the walnut interspecies hybrid transcriptome, and provides extensive new insights into gene boundaries, tissue-specific transcriptional profiles, and transcriptional regulation of VC activity related to grafting. The findings will not only supplement the existing walnut omics database, but also provide a valuable resource for further studies of heterosis and functional genomics research in walnut and other woody plants.

## Data Availability

The datasets generated for this study can be found in NCBI, https://www.ncbi.nlm.nih.gov/sra/PRJNA506793.

## Author Contributions

DP designed and supervised the study. QM wrote the manuscript, and carried out the experiments and data analysis. DB conducted the bioinformatics analysis. JZ collected the sample, evaluated the conclusions, and supervised the experiments. YW revised the manuscript and supervised the bioinformatics analysis. All authors read and approved the final version of the manuscript.

## Conflict of Interest Statement

The authors declare that the research was conducted in the absence of any commercial or financial relationships that could be construed as a potential conflict of interest.

## Footnotes

1 https://figshare.com/articles/Assembled_sequences_and_sequence_annotation_for_Juglans_hybrid/8197619

2 http://www.usadellab.org/cms/?page=trimmomatic

3 ftp://ftp.ccb.jhu.edu/pub/dpuiu/Walnut/English_walnut/v2.0d/

4 https://ftp.ncbi.nlm.nih.gov/refseq/release/

5 http://trinotate.github.io/

6 http://www.arabidopsis.org/tools/bulk/go/index.jsp

7 http://www.genome.jp/kegg/kaas/

8 ftp://ftp.ccb.jhu.edu/pub/dpuiu/Walnut/English_walnut/v2.0d/

## Supplementary Material

FIGURE S1 Size distribution of ‘ZNS’ transcript length and multiple-isoform transcripts.

FIGURE S2 Abundance of unigenes expressed specifically in Root validated by relative qRT-PCR. Curves showing the expression level of transcriptomic sequencing while bars showing the results of qRT-PCR.

FIGURE S3 Heatmap of differentially expressed genes (logFC > 1.5) involved transcription factors, defense response, age-dependent, cell division, cytoskeleton, cell wall, and transport. The bar represents the scale of the expression levels for each gene (TMM) in the MS and JS as indicated by red/green rectangles. Red rectangles indicate up-regulation of genes and green represents down-regulation. VC: vascular cambium; MS: new shoots from ‘ZNS’ adult seedling tree; JS: new shoots from ‘ZNS’ grafted tree.

FIGURE S4 Expression patterns for genes involved the plant hormone signal transduction pathway (Ko04075). TMM: trimmed mean of M values. Red, green and grey squares showing up-regulated, down-regulated and undetected genes, separately. Red bar shows the sample of new shoots from ‘ZNS’ grafted tree (JS) and blue bar shows new shoots from ’ZNS’ adult seedling tree (MS).

FIGURE S5 Expression patterns for differentially expressed genes in the cell cycle pathway (ko04110). TMM: trimmed mean of M values. Red, green and grey squares showing up-regulated, down-regulated and undetected genes, separately. Red bar shows the sample of new shoots from ‘ZNS’ grafted tree (JS) and blue bar shows new shoots from ‘ZNS’ adult seedling tree (MS).

TABLE S1 Statistics of the completeness of ‘ZNS’ transcriptome based on 248 core eukaryotic genes by the software CEGMA. Prots–number of 248 ultra-conserved CEGs present in genome. Completeness (%)–percentage of 248 ultra-conserved CEGs present. Total–total number of CEGs present including putative orthologs. Average–average number of orthologs per CEG. Ortho (%)–percentage of detected CEGS that have more than 1 ortholog. Complete–predicted proteins in the set of 248 CEGs that, when aligned to the HMM (a hidden markov model) for the KOG (eukaryotic orthologous groups) for that protein family, give an alignment length that is at least 70% of the protein length. Partial–If a protein is not complete, but exceeds a pre-computed minimum alignment score, then we call the protein ‘partial’.

TABLE S2 Blast result against the Nr database.
