# Supplementary figures and images for: The Transcriptome Landscape of Walnut Interspecies Hybrid (Juglans hindsii × Juglans regia) and Regulation of Cambial Activity in Relation to Grafting
Source: Front Genet. 2019 Jun 21;10:577. doi: 10.3389/fgene.2019.00577 (PMC6598599; doi:10.3389/fgene.2019.00577)

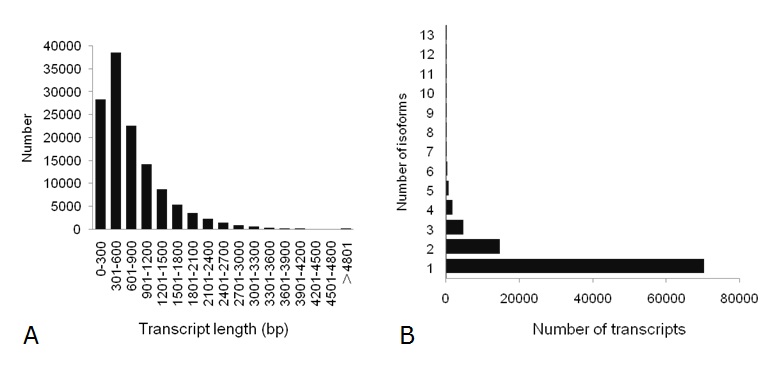

Supplement: FIGURE S1 — Size distribution of ‘ZNS’ transcript length and multiple-isoform transcripts. [file Image_1.JPEG]

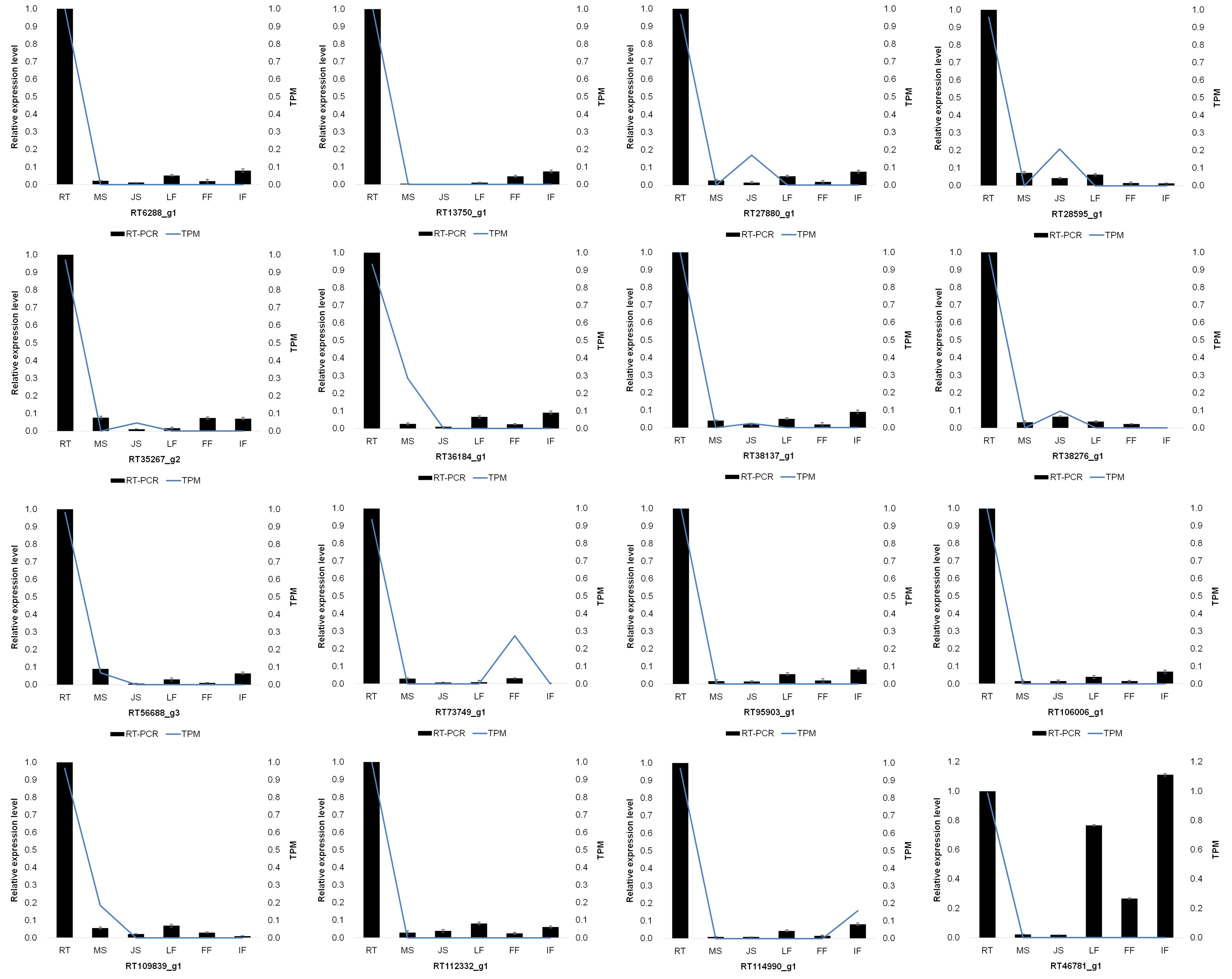

Supplement: FIGURE S2 — Abundance of unigenes expressed specifically in Root validated by relative qRT-PCR. Curves showing the expression level of transcriptomic sequencing while bars showing the results of qRT-PCR. [file Image_2.JPEG]

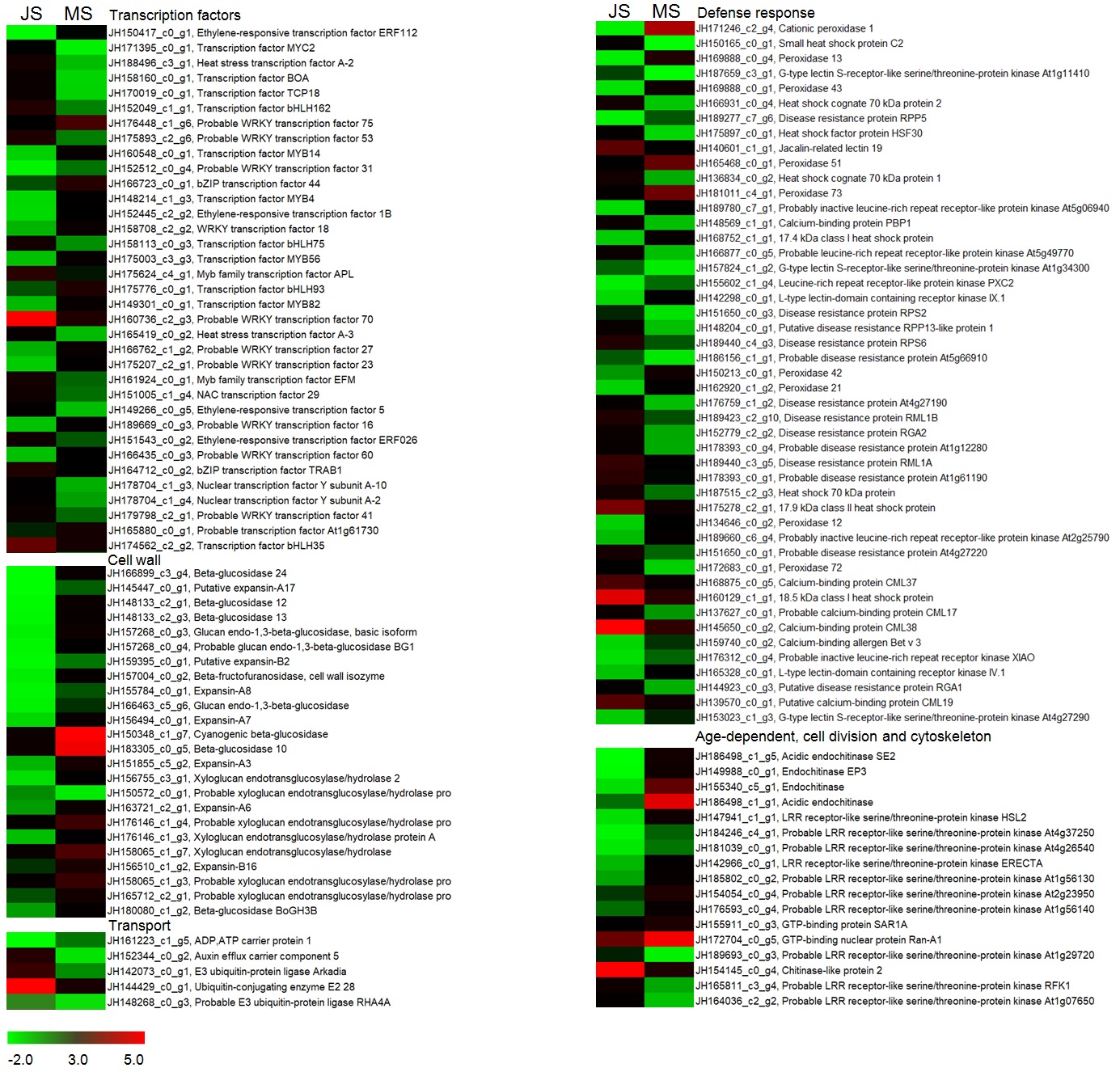

Supplement: FIGURE S3 — Heatmap of differentially expressed genes (logFC > 1.5) involved transcription factors, defense response, age-dependent, cell division, cytoskeleton, cell wall, and transport. The bar represents the scale of the expression levels for each gene (TMM) in the MS and JS as indicated by red/green rectangles. Red rectangles indicate up-regulation of genes and green represents down-regulation. VC: vascular cambium; MS: new shoots from ‘ZNS’ adult seedling tree; JS: new shoots from ‘ZNS’ grafted tree. [file Image_3.JPEG]

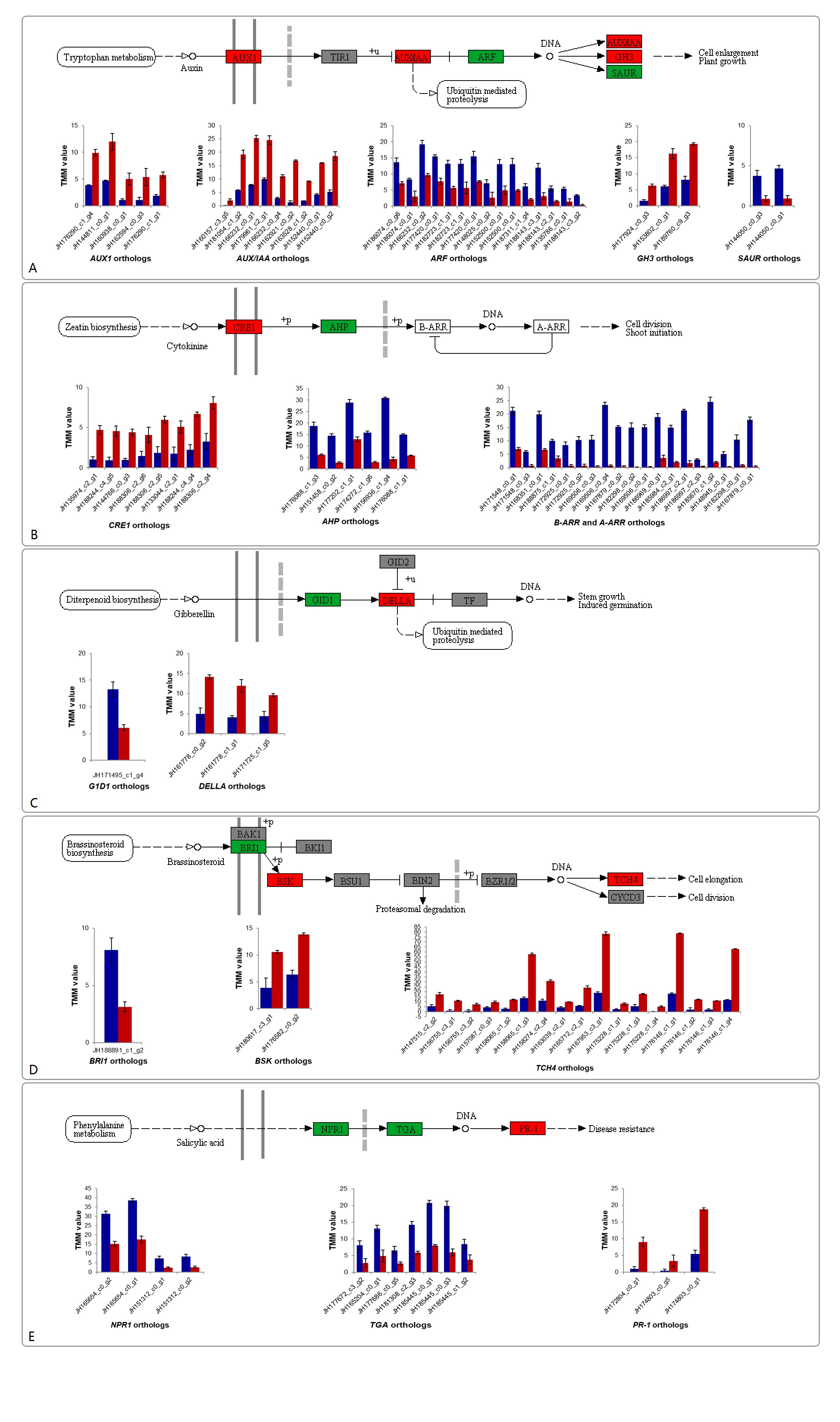

Supplement: FIGURE S4 — Expression patterns for genes involved the plant hormone signal transduction pathway (Ko04075). TMM: trimmed mean of M values. Red, green and grey squares showing up-regulated, down-regulated and undetected genes, separately. Red bar shows the sample of new shoots from ‘ZNS’ grafted tree (JS) and blue bar shows new shoots from ’ZNS’ adult seedling tree (MS). [file Image_4.JPEG]

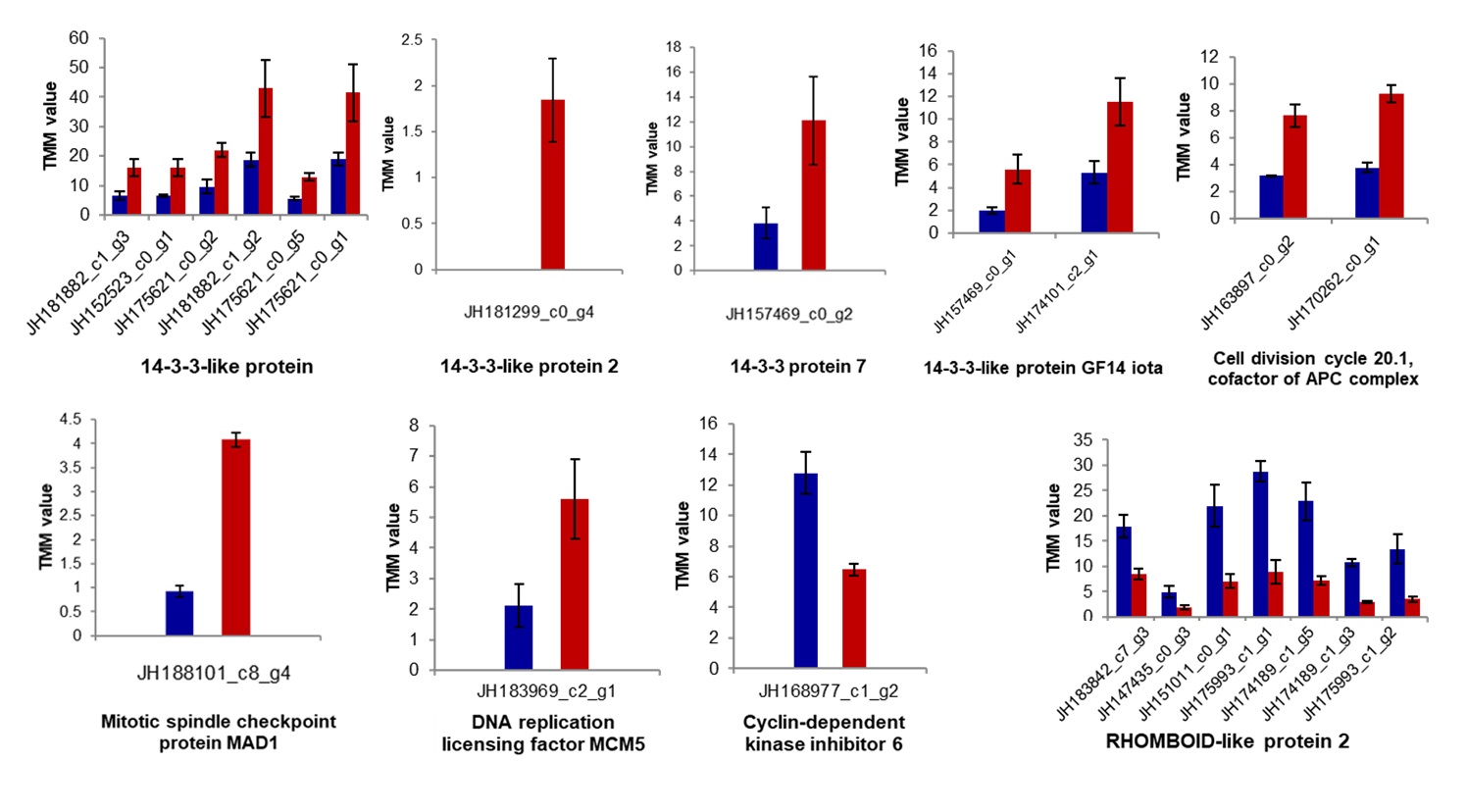

Supplement: FIGURE S5 — Expression patterns for differentially expressed genes in the cell cycle pathway (ko04110). TMM: trimmed mean of M values. Red, green and grey squares showing up-regulated, down-regulated and undetected genes, separately. Red bar shows the sample of new shoots from ‘ZNS’ grafted tree (JS) and blue bar shows new shoots from ‘ZNS’ adult seedling tree (MS). [file Image_5.JPEG]
